# Climate Finance and Youth NEET in Africa: Cross-Country Evidence from 46 Nations

**DOI:** 10.1038/s41598-026-54358-7

**Published:** 2026-05-21

**Authors:** Jeremy Ko, Arsene Mouongue Kelly, Chun Kai Leung, Chuangjian Xin, Boris Lok-Fai Pun, Anthony Chun-Yue Cheung, Mohammad Ridwan, Harry F. Lee

**Affiliations:** 1 Center for Comparative and International Studies, ETH Zurich, Zurich, Switzerland; 2 Center for Studies and Research in Economics and Management (CERME), University of Dschang, Dschang, Cameroon; 3 Global Society and Sustainability Lab, Faculty of Social Sciences, The University of Hong Kong, Hong Kong, China; 4 Institute for the Humanities and Social Sciences, The University of Hong Kong, Hong Kong, China; 5 Faculty of Business and Economics, The University of Hong Kong, Hong Kong, China; 6 School of Finance, Shanghai University of International Business and Economics, Shanghai, China; 7 School of Governance and Policy Science, The Chinese University of Hong Kong, Hong Kong, China; 8 Hong Kong Asia-Pacific Institute of Studies, The Chinese University of Hong Kong, Hong Kong, China; 9 Friends of the Earth (Hong Kong), Hong Kong, China; 10 Department of Economics, Noakhali University of Science and Technology, Sonapur , Bangladesh; 11 Department of Geography and Resource Management, The Chinese University of Hong Kong, Hong Kong, China; 12 Institute of Environment, Energy and Sustainability, The Chinese University of Hong Kong, Hong Kong, China; 13 Center for Green Finance and Sustainability Studies, International Islamic University Chittagong, 4318 Chattogram, Bangladesh

**Keywords:** Climate finance, NEETs, Human capita, Youth employment, African countries, Climate change, Climate sciences, Environmental social sciences

## Abstract

This paper examines whether climate finance contributes to reducing youth inactivity, measured by the share of young people not in education, employment, or training (NEET), across 46 African countries from 2011 to 2021. Drawing on Human Capital Theory, the study conceptualizes climate finance as an external investment that can expand capabilities, enhance skills, and strengthen labor-market inclusion when effectively absorbed by domestic institutions. Using panel data and the Driscoll–Kraay corrections as well as the Instrumental Variables Generalized Method of Moments (IV–GMM) techniques, the analysis finds a consistent and statistically significant negative association between total climate‑finance inflows and youth NEET rates. This relationship remains robust across multiple model specifications, extended socio‑economic controls, and heterogeneity tests. The findings suggest that climate finance functions not only as an environmental mechanism but also as a developmental tool, promoting youth engagement through human capital formation and skill development. Policy‑wise, aligning climate‑finance frameworks with national education, training, and employment systems can potentially transform external funds into effective catalysts for reducing NEET rates.

## Introduction

 Africa remains one of the most vulnerable continents to the destabilizing effects of climate change, experiencing a complex interplay of environmental pressures and socio-economic fragility that threatens livelihoods and development gains^1^. Rising temperatures, recurrent droughts, and climate‑related disasters have undermined key livelihood systems such as agriculture, fisheries, and forestry, which together employ more than 55% of the working population^2,3^. The IMF estimates that climate‑induced losses cost up to 5% of GDP annually in many Sub‑Saharan African economies^4^, while the African Development Bank^5^ warns that Africa will need to invest over USD 3 trillion in mitigation and adaptation by 2030 to implement its Nationally Determined Contributions (NDCs). These pressures have compounded existing structural weaknesses, limited infrastructure, low industrial diversification, and fragile institutions, which constrain sustainable and inclusive growth.

In parallel with these environmental challenges, Africa faces a structural economic dilemma rooted in its unprecedented youth bulge. The continent’s 226 million people aged 15–24 as of 2015 already constitute the world’s largest and fastest‑growing youth cohort, projected to reach nearly 500 million by 2080^6^. This demographic expansion, often described as a potential demographic dividend, has unfolded alongside stagnant formal‑sector growth and low employment elasticity, generating persistent mismatches between labor supply and demand. Across the region, about 21.9% of youth are not in education, employment, or training (NEET), a modest improvement from 22.4% before the COVID‑19 pandemic but still above the global average of 20.4%^7^. In several regions, particularly North and Southern Africa, NEET rates exceed 30%, reflecting enduring structural barriers, including limited industrial diversification, weak vocational systems, and the dominance of informal and subsistence sectors^8^. This macro‑structural imbalance between rapid demographic growth and slow economic transformation constrains productivity, strains public services, and heightens the risk of exclusion and instability. Consequently, tackling youth inactivity is not merely a labor-market concern but a foundational condition for inclusive and sustainable development, as emphasized in Sustainable Development Goal 8 (Decent Work and Economic Growth).

Within this demographic and structural context, exploring financing mechanisms that address environmental risks while enabling labor‑market opportunities has become increasingly relevant. One such mechanism is climate finance, financial resources mobilized from public, private, and multilateral sources to support mitigation and adaptation projects^2,9^. Beyond its environmental objectives, climate finance can influence economic structures by supporting green investment, climate‑resilient infrastructure, and skill‑intensive projects in renewable energy and sustainable agriculture. Over the past decade, climate‑finance inflows to Africa have expanded from about USD 3.4 billion in 2011 to nearly USD 30 billion in 2021 (OECD, 2025). Yet despite this growth, their social and labor-market impacts remain underexplored. Existing research has largely focused on macroeconomic and environmental outcomes, such as emissions reductions, energy transitions, and poverty alleviation^10–12^, while overlooking how these financial inflows shape employment structures and labor inclusion. In particular, the effects on young people, who represent Africa’s fastest‑growing workforce segment, remain virtually absent from the literature. To the best of our knowledge, no cross‑national study to date has examined whether rising climate‑finance inflows influence the proportion of youth not in education, employment, or training (NEET), despite this indicator’s relevance as a measure of economic participation and social stability.

Against this backdrop, this paper helps fill these gaps by linking climate‑finance inflows directly to youth labor-market outcomes across 46 African countries from 2011 to 2021. Guided by Human Capital Theory^13,14^, the study conceptualizes climate finance as a potential external investment that can enhance capabilities, skills, and employment opportunities when effectively absorbed into domestic systems. Employing a two‑way fixed‑effects panel approach with Driscoll–Kraay corrections and IV–GMM estimation, the analysis provides the first region‑wide empirical evidence on whether climate‑related funding corresponds with lower youth NEET rates. By situating employment at the center of the inquiry, the paper reframes climate finance as a mechanism that can reinforce both environmental sustainability and inclusive human‑capital development in Africa.

The remainder of this paper is organized as follows. Section 2 reviews theoretical and empirical literature and formulates the hypotheses. Section 3 describes the data, variables, and econometric approach. Section 4 presents and discusses the empirical findings, and Sect. 5 concludes with policy implications and directions for future research.

## Literature review

### Human capital theory

Human Capital Theory conceptualizes education, training, and knowledge accumulation as productive investments that enhance individual capabilities and aggregate economic performance. The foundational contributions of Schultz^13^ and Becker^14^ reframed economists’ understanding of labor markets by treating skills and knowledge not as fixed attributes but as assets that can be systematically developed. In this view, individuals who invest time and resources in education increase their future productivity and earning potential, while societies that prioritize workforce development generate the conditions for technological progress and sustain economic growth. The theory’s core insight profoundly shaped both labor economics and development policy by showing that human capabilities respond to investment much like physical capital.

Later extensions incorporated these micro-level foundations into endogenous growth frameworks. Lucas^15^ and Romer^16^ showed that economies with more human capital tend to have greater innovation, better knowledge spillovers, and faster structural change. The mechanism operates cumulatively: education and skill formation raise worker productivity, thereby expanding adaptive capacity and enabling endogenous technological change. Empirical evidence from Mincer^17^ and Psacharopoulos^18^ consistently documents substantial private and social returns to schooling across diverse contexts, reinforcing the proposition that human capital investment generates durable productivity gains.

Within this human capital framework, climate‑finance inflows can be understood as exogenous investments that enhance the stock and quality of human capital by shaping labor‑market dynamics in recipient economies. Although conceived primarily for environmental objectives, such inflows often fund activities including technology transfer, innovation diffusion, and workforce training that strengthen the knowledge and skill base required for sustainable production^19^. By expanding opportunities for education and experience in climate‑related sectors, these investments increase workers’ productivity and employability, generating new avenues for decent work and facilitating the transition from low‑productivity to higher‑value green employment. In theoretical terms, climate finance serves as a complementary input into the human capital accumulation process, enabling recipients to convert financial resources into capabilities that sustain innovation and adaptive growth^20^. Employment effects emerge not merely from job creation but from the upgrading of workforce competencies, which link climate action to the central tenet of Human Capital Theory that investment in people is the foundation of long‑term economic and social transformation.

### Existing research on climate finance in Africa

Research on climate finance in Africa has progressively shifted from a descriptive mapping of financial flows to more analytical inquiries into the economic, institutional, and social mechanisms that determine the effectiveness of development outcomes. Rather than viewing climate finance as a uniform input for climate mitigation and adaptation, recent empirical studies highlight that its impact is relational, conditioned by governance capacity, institutional quality, and the structural characteristics of recipient economies.

One prominent line of inquiry examines the determinants of allocation and their developmental implications. Doku et al.^9^ show that countries with larger populations, weaker fiscal capacity, and stricter rule of law tend to receive greater climate-finance inflows. This pattern reflects a tension intrinsic to Africa’s climate‑finance landscape: resources are directed toward countries exhibiting high vulnerability yet limited absorptive ability, reflecting a balancing act between need and fiduciary reliability. Extending the discussion from allocation to developmental performance, Phiri and Doku^21^ and Doku, Richardson, and Essah^22^ find that while climate finance improves food availability, it does little to enhance food access, stability, or utilization. Together, these studies suggest that the effectiveness of climate finance depends less on funding volumes and more on institutional coherence and policy integration. That is, the ability of domestic systems to translate financial inputs into productivity and inclusion.

Related research connects these allocative and institutional dynamics to environmental performance. Doku, Ncwadi, and Phiri^23^ identify an inverted‑U EKC relationship among climate finance, income, and emissions: at lower development levels, additional finance may initially increase emissions, but beyond a threshold of USD 910 million to 1.6 billion, further inflows contribute to mitigation. Most African economies, however, remain below this critical scale. This finding aligns with evidence from Lee et al.^24^ and Gong et al.^25^, who document that mitigation finance achieves stronger results in high‑capacity systems but diminishing returns in resource‑dependent contexts. Collectively, these analyses converge on a central conclusion: the environmental and economic outcomes of climate finance are inseparable from structural transformation and institutional strengthening.

Building on this insight, scholars have increasingly examined how climate finance interacts with broader systems of provision such as agriculture, energy, and social protection. Phiri and Doku^21^ and Kelly^26^ show that climate‑related investments stimulate agricultural productivity but rarely enhance nutritional resilience or equitable access, outcomes heavily dependent on market governance and state capacity. Within the energy sector, Borojo et al.^27^ demonstrate that countries possessing stronger administrative frameworks translate climate finance more effectively into renewable‑energy production. Similarly, Njangang^28^ reports that mitigation‑oriented flows yield greater reductions in energy vulnerability than adaptation finance, though the benefits vary sharply across income groups. These complementary studies point to a shared mechanism: climate finance drives transformation most effectively when embedded in contexts with robust managerial capacity and coherent policy institutions.

Other contributions have emphasized the distributional and equity dimensions of climate‑finance use. Wang and Njangang^29^ observe that where governance deficits persist, climate‑finance inflows can entrench elite capture and corrupt appropriation, undermining climate goals. Wong^30^ further illustrates how gendered and generational inequalities embedded in land and decision‑making systems constrain equitable access to climate‑finance benefits. Addressing such structural constraints, Kelly and Ndeffo^3^ contrast the immediate behavioral impact of environmental taxation with the slower but potentially deeper structural reforms supported by climate finance, arguing that sustained institutional improvement is crucial for long‑term effectiveness. Expanding the evaluative lens, Kelly^2^ finds that climate finance enhances subjective well‑being through ecological restoration and improved institutional legitimacy, suggesting that the benefits of finance extend beyond purely economic measures to societal cohesion and trust. As such, the literature positions climate finance in Africa as a conditional catalyst: its success depends less on funding size than on the fit between external capital and domestic capability.

### Climate finance and Socio-economic impacts

Building on the institutional dynamics outlined earlier, recent research broadens the focus from allocation and governance to the socio‑economic consequences of climate finance. No longer viewed purely as a fiscal input for green growth, it is increasingly analyzed as a developmental mechanism that reorganizes labor, industrial structures, and welfare systems in context‑specific ways. Comparative evidence across developing regions reveals a dual pattern: climate finance can expand productive opportunities while reinforcing pre‑existing social and structural inequalities. Zhao, Li, and Hou^31^ find that mitigation‑focused projects deepen gender disparities across ninety‑two developing economies, particularly in Africa, where capital‑intensive sectors remain male‑dominated and social norms limit women’s access to climate‑related employment. In contrast, Mohan and Morris^32^ show that climate‑finance inflows stimulate entrepreneurship and small‑business formation, with strong growth in emerging green sectors. Together, these studies underline the socio‑economic duality of climate finance: it can foster innovation and enterprise but may also reproduce inequality where labor markets and institutional frameworks remain rigid.

At the productive and industrial levels, the transformative effects of climate finance vary across contexts. Evidence from China, such as Xin et al.^33^ and Fu et al.^34^, shows that the Green Finance Reform and Innovation Pilot Zones have encouraged labor reallocation from emission‑intensive manufacturing toward cleaner, service‑oriented, and high‑technology sectors. These policies produce short‑term adjustments within firms but lead to longer‑term employment and productivity gains, illustrating how sustained green‑finance frameworks can promote structural diversification. In contrast, many African economies direct climate finance toward primary production and extractive industries, where it supplements existing activities rather than driving technological upgrading^35^, thereby limiting their potential for sustainable economic growth and innovation relative to outcomes observed in China. This comparison highlights how the interaction between climate finance, domestic innovation capacity, and industrial policy shapes economic outcomes.

The literature on energy access, inequality, and poverty also emphasizes the role of absorptive capacity and governance. Using multi‑country data, Njangang, Padhan, and Tiwari^28^ find that mitigation‑oriented finance reduces energy vulnerability more effectively than adaptation finance, particularly in low‑ and middle‑income economies. Complementary evidence from China by Zhao, Wang, and Dong^36^ shows that green finance alleviates energy poverty directly and indirectly through technological innovation and industrial upgrading, though benefits are concentrated in more advanced eastern provinces. In Sub‑Saharan Africa, Doku^12^ finds that climate finance contributes to growth and poverty reduction but has limited effects on inequality, while Li et al.^37^ observe that countries with stronger governance convert mitigation finance into greater equity gains. Likewise, Xu et al.^38^ further show that in China, industrial upgrading and regional adaptation mediate the link between green finance and poverty reduction. Collectively, these studies suggest that the social inclusiveness of climate finance is closely associated with governance quality and the capacity to integrate financial flows into wider development strategies.

Beyond economic outcomes, climate finance has important implications for human development, social protection, and resilience systems. Gong et al.^25^ find that both mitigation and adaptation flows raise the Human Development Index across developing countries by improving education, health, and living conditions, with effects strongest in lower‑income but institutionally organized states. Aleksandrova, Kuhl, and Malerba^39^ report that the Green Climate Fund has begun incorporating social‑protection frameworks into climate programs, with notable results for rural livelihoods and climate‑service initiatives but weaker progress in urban and housing projects. In fragile environments such as Somalia, Nor^40^ shows that climate‑sensitive development finance helps strengthen rural resilience and reduce skilled out‑migration by investing in adaptive livelihoods. Together, these works highlight that climate finance contributes not only to economic performance but also to broader welfare and stability objectives. The evidence across these contexts shows that climate finance serves as both an economic and social instrument. In China and other emerging economies, it supports technological upgrading, industrial diversification, and measurable welfare gains. In Africa and other lower‑readiness regions, climate finance continues to provide growth and adaptive capacity but remains constrained by sectoral concentration and uneven institutional integration, which limits its effectiveness in addressing the diverse challenges posed by climate change in these areas.

### Literature gap and research contribution

Although research on climate finance in Africa has advanced significantly, much of it continues to frame environmental funding in macroeconomic or ecological terms, focusing on outcomes such as emissions reductions, energy transitions, or resource efficiency^21,23,27^. What remains insufficiently examined is how climate‑finance inflows engage with the labor-market dimension of development, specifically, whether increased funding corresponds with broader, more inclusive employment structures. Existing studies have established that climate finance influences production systems and sectoral performance, yet they largely stop short of assessing its implications for labor demand, job composition, or human capital formation. Much of the related empirical work has been concentrated outside Africa, particularly in country-specific contexts such as China, where analyses have linked green finance to energy poverty alleviation, industrial upgrading, and labor reallocation [e.g., 35, 36, 38, 40]. While these studies provide valuable comparative insights into the potential developmental effects of sustained climate‑finance frameworks, their findings remain context‑dependent and are not easily transferable to regions with weaker institutional structures, larger informal labor markets, and different demographic profiles. Even research addressing broader socio‑economic co‑benefits^25,3,37^ tends to treat employment outcomes as secondary or incidental to environmental performance. Consequently, the relationship between climate finance and employment remains empirically underspecified, and there is limited evidence on whether the expansion of climate‑related investment improves job availability, job quality, or labor-market dynamism. This omission constrains understanding of how international environmental resources intersect with domestic systems of labor allocation and skill development, both of which are crucial for translating financial inflows into sustainable and inclusive economic transformation in African economies.

Second, despite Africa’s rapidly growing youth population, little research considers generational specificity within the climate‑finance–employment nexus. Existing studies highlighting poverty, gender, or inequality^12,30,31^ do not treat young people as a distinct analytical category, even though youth constitute the majority of those entering labor markets most exposed to climate risks. To the best of our knowledge, no empirical work has yet examined, on a cross‑national basis, the relationship between external climate‑finance inflows and indicators of youth engagement, such as NEET rates, which capture simultaneous exclusion from education, employment, and training and thereby provide a multidimensional view of youth vulnerability. Unlike conventional unemployment measures that track only those actively seeking work, the NEET indicator reflects deeper structural constraints, including limited access to education, weak vocational systems, and discouraged labor participation, which define the realities of many African youths. This omission is particularly significant from the perspective of Human Capital Theory, which posits that investment, whether in education, skills, or enabling economic systems, enhances individual productivity and collective welfare^13,14^. If climate finance acts as a form of exogenous investment that builds skills, strengthens adaptive capacities, and supports labor‑absorptive sectors, its human‑capital effects should be most evident in reductions in NEET rates, where improved access to training, productive employment, and inclusive participation signals enhanced capability formation. Given that young people constitute the largest share of Africa’s working‑age population and represent the foundation of its prospective demographic dividend, analyzing NEET dynamics provides a theoretically grounded and empirically precise lens for assessing whether climate‑finance inflows foster inclusive, youth‑oriented development across the continent.

Building on these gaps, this paper makes three main contributions to the literature on climate finance and development. First, it explicitly introduces the labor-market perspective into the analysis of climate-finance impacts, interpreting external environmental funding not only as an ecological or fiscal input but also as a potential catalyst for employment and human-capital formation. Second, it provides the first cross‑national empirical assessment linking climate‑finance inflows to youth labor‑market outcomes, operationalized through NEET rates, across 46 African countries from 2011 to 2021. By situating youth inactivity at the center of analysis, the study broadens the scope of climate‑finance research to capture intergenerational and demographic dynamics largely absent from prior scholarship. Third, the paper strengthens methodological rigor in this field through a two‑way fixed‑effects design with Driscoll–Kraay corrections and IV–GMM estimation, supported by extensive robustness and heterogeneity tests, to deliver credible region‑wide evidence. These contributions extend existing research beyond macroeconomic and environmental evaluation toward a clearer understanding of how climate‑finance inflows intersect with Africa’s structural employment landscape and the human‑capital foundations of inclusive growth.

### Mechanism and hypothesis

HCT provides a framework for interpreting the developmental role of climate finance. At its core, the theory views education, training, and knowledge accumulation as productive investments that enhance individual capability and expand an economy’s adaptive capacity^13,14^. Just as investment in physical capital improves productivity, investment in human capital strengthens the stock of skills, innovation, and institutional efficiency that underpin sustainable growth. Climate finance aligns with this logic as an external source of investment in capability formation. Although conceived primarily to support mitigation and adaptation goals, the effective implementation of climate‑finance projects demands technical proficiency in areas such as renewable‑energy systems, sustainable‑agriculture techniques, climate‑risk modeling, and infrastructure design. The financial and technological resources mobilized through these initiatives, therefore, stimulate the formation and upgrading of human capital by expanding professional expertise, vocational competence, and problem‑solving capacity within the labor force^34^. Through this investment logic, climate‑finance inflows contribute to building a more skilled and adaptive workforce, yielding developmental returns that extend from environmental resilience to productivity and employment.

Within this HCT framework, the connection between climate finance and youth NEET outcomes is understood through the employment‑enabling role of human‑capital formation. As external financial inflows enhance the stock and relevance of human capital, they strengthen young people’s preparedness for work by equipping them with technical, managerial, and problem‑solving competencies demanded in evolving labor markets. Youths in Africa, who typically face entry barriers due to limited experience and skill misalignment^41^, benefit when climate‑related investments create training opportunities, apprenticeship programs, and transferable skills in green and adaptive sectors. A more capable and adaptable youth workforce fosters smoother transitions from schooling into work, raises the returns to participation, and reduces the economic and structural drivers of inactivity^39,42^. In this sense, climate finance functions as a developmental investment that reinforces the employability conditions described by Human Capital Theory, linking capability enhancement directly to greater youth inclusion in education, training, and productive employment. Consequently, lower NEET rates represent one observable outcome of human‑capital advancement derived from the effective absorption of climate‑finance resources. The study, therefore, posits the following hypothesis:

#### H₁

*Climate finance is associated with a reduction in youth NEET rates across African countries.*

## Material and Method

### Study focus and temporal scope

This study focuses on 46 African countries (see full list in Table A1), a region characterized by high exposure to climate variability and persistent challenges of youth underemployment. The structural interaction among environmental stress, demographic pressure, and limited labor‑market absorption makes Africa a critical context for examining whether climate finance operates not only as an ecological intervention but also as a mechanism to expand economic opportunities for youth. In many countries across the region, formal employment remains constrained by infrastructural limitations, rapid population growth, and the long‑term effects of uneven development planning^3,43^.

The analysis covers the period 2011–2021, selected based on both data availability and substantive relevance. The NEETs indicator, used as the dependent variable, is consistently available only from 2011 onward, while disaggregated climate finance data become increasingly incomplete after 2021. Following established practices in climate‑finance research^35^, this timeframe enables the use of comprehensive, high‑quality datasets from authoritative sources such as the OECD. The sample excludes a small number of African countries, including Somalia, South Sudan, and Eritrea, due to incomplete or inconsistent data on NEETs or climate‑finance disbursements during the study period. Despite these omissions, the sample provides broad and representative coverage of the continent’s economic and regional diversity. This decade captures a formative period in which global climate‑finance commitments expanded significantly, accompanied by increasing policy emphasis on youth inclusion in development strategies. It also spans major international debates on transition justice, adaptive governance, and the developmental co‑benefits of climate investment, offering a relevant context for investigating the links between climate finance and youth employment outcomes across African economies.

### Presentation of variables

#### Dependent variable

The dependent variable in this study is the NEET rate, the percentage of young people aged 15 to 24 who are not in education, employment, or training. This measure captures youth disengagement from both the labor market and the educational system, offering a broader understanding of exclusion than conventional unemployment rates, which count only active job seekers. By including those who are discouraged or outside formal education and training, the NEET rate more accurately reflects the scale of youth vulnerability and the capacity of economies to integrate their young populations into productive activities. The 15–24 age group follows the international standard used by institutions such as the International Labor Organization (ILO) and the United Nations, representing the key transition period from schooling to work. However, this definition has certain limitations. In many countries, legal restrictions and continued education requirements may prevent younger individuals, particularly those aged 15 to 17, from fully participating in the labor market^44^. As a result, some portion of the NEET population within this cohort could reflect institutional constraints rather than voluntary inactivity or disengagement.

Even so, this age bracket remains particularly suitable in the African context. In many African economies, labor‑market entry tends to occur earlier than in high‑income regions due to limited access to higher education and the economic necessity for young people to begin working at younger ages^44^. Consequently, being “not in education, employment, or training” between ages 15 and 24 often signals genuine exclusion from both systems rather than a temporary or normative stage of dependency. Moreover, this period encapsulates the crucial school‑to‑work transition, when structural and institutional constraints, such as labor‑market informality, limited skill development, and weak governance, most strongly influence youth outcomes^45^. Overall, the NEET rate provides a theoretically grounded measure of youth labor‑market vulnerability. It enables the analysis to capture both economic and educational dimensions of exclusion, which are particularly relevant in African economies, where early school leaving and constrained employment opportunities remain defining features of youth labor‑market challenges.

#### Independent variables

The main explanatory variable in this study is logged total climate finance, defined as the natural logarithm of the combined value of adaptation and mitigation finance received annually by each African country. This measure represents the aggregate volume of financial resources directed toward climate‑related activities at the country‑year level, expressed in thousands of current USD. The logarithmic transformation is applied to correct for skewness in the distribution of financial flows, as some countries receive disproportionately large disbursements, and to facilitate the interpretation of estimated coefficients in percentage terms. Data on climate finance are obtained from the OECD Development Assistance Committee (OECD‑DAC) Creditor Reporting System (CRS), which reports disbursements from bilateral and multilateral sources in a recipient–donor format. Following established practice^2,46^, these data were aggregated into a country‑year panel by summing all climate‑related inflows received by each African country in a given year. Using the OECD‑DAC data ensures methodological consistency and cross‑country comparability over time. It should be noted, however, that climate finance is measured in thousands of current USD without adjustment for purchasing power parity (PPP) or domestic currency fluctuations. While this approach aligns with most empirical studies using global datasets, it may introduce minor comparability biases across countries with different price levels and exchange‑rate regimes, particularly affecting the accuracy of financial assessments in nations with volatile currencies or high inflation. Nonetheless, the choice reflects a practical trade‑off between data completeness and methodological consistency, ensuring a uniform basis for measuring climate‑related financial inflows across the sample.

#### Control variables

A theoretically grounded set of control variables is incorporated to more precisely isolate the relationship between climate finance and youth NEET outcomes, ensuring that the estimated effects reflect genuine variation in climate financing rather than underlying macro‑structural, demographic, or institutional differences. These covariates capture the broader socioeconomic environment that shapes young people’s opportunities for education, training, and employment in African labor markets.

- **Economic development**, measured by *GDP per capita*, reflects both productive capacity and structural transformation. According to human capital and modernization theories, higher levels of development are generally associated with improved labor demand, expanded educational opportunities, and stronger state capacity to invest in youth employment programs, all of which can reduce the NEET share^47^. Conversely, low development levels tend to limit productive job creation and reinforce informal or subsistence employment.
- **Population (log)** captures demographic pressures on labor markets. From a labor supply perspective, a rapidly growing youth population can intensify competition for limited jobs and social services, increasing the likelihood of youth inactivity. When such expansion outpaces job creation (“youth bulge”), structural unemployment and NEET rates rise due to a mismatch between labor supply and absorption capacity^48^.
- **Urbanization**, measured as the share of the population residing in urban areas, reflects the spatial concentration of economic activity and access to services. Higher levels of urbanization are expected to reduce youth NEET rates by expanding exposure to formal education systems, training institutions, and a broader range of employment opportunities in urban labor markets, thereby enhancing young people’s chances of labor market integration^49,50^.
- **Labor Freedom Index** captures the degree of regulatory flexibility in the labor market, encompassing hiring practices, wage policies, and contractual arrangements. Greater labor freedom is theoretically expected to reduce youth NEET rates by lowering barriers to job entry, encouraging firm-level hiring, and fostering an environment conducive to entrepreneurship and private-sector participation, thereby improving young people’s integration into the labor market^51^.
- **Democracy Index** serves as a measure of the inclusiveness and accountability of political institutions. Higher levels of democracy are theoretically expected to lower youth NEET rates by fostering responsive governance, improving policy implementation, and promoting greater investment in education, training, and labor programs that support youth participation in the workforce^52^.
- **Political Corruption** captures inefficiencies and distortions in governance. Institutional economics emphasizes that corruption undermines policy credibility, deters private investment, and diverts resources from productive and employment‑oriented spending. In such contexts, youth are more likely to remain excluded from labor markets due to weakened institutional support and unequal access to opportunity^53^.

Taken together, these covariates embody complementary theoretical perspectives from human capital development, demographic transition, urban and institutional economics, and governance theory. Their inclusion provides a conceptually coherent and empirically robust framework for analyzing youth NEET outcomes, ensuring that the estimated relationship between climate finance and labor‑market engagement is properly conditioned on the fundamental structural and institutional factors that shape youth economic inclusion across African countries.

**Table 1:** Descriptive Statistics.

Variable	Description	Observations	Mean	Std. Dev.	Min	Max
Climate finance (log)	Logged total climate finance (combined adaptation and mitigation) received annually by each country (in thousands of USD), from public and non-governmental sources.	460	5.578	5.602	−6.908	13.442
Mitigation finance (log)	Logged total climate mitigation finance received annually by each country (in thousands of USD), from public and non-governmental sources.	460	4.186	5.896	−6.908	12.684
Adaptation finance (log)	Logged total climate adaptation finance received annually by each country (in thousands of USD), from public and non-governmental sources.	460	4.371	6.104	−6.908	12.971
NEET	The percentage of young people not in education, employment, or training” measures the share of individuals aged 15 to 24 years who are neither employed nor engaged in any form of education or training.	460	22.193	8.756	5.07	41.837
Economic development	GDP per capita in current USD (log-transformed).	460	7.343	0.972	5.347	9.86
Population (log)	Logged national population (*de facto*) per year.	460	2.172	1.597	−2.408	5.363
Urbanization	Proportion of the population living in urban areas.	460	45.294	18.205	11.19	90.42
Labor Freedom Index	Composite index (0–100) based on regulatory flexibility, hiring practices, working hours, dismissal procedures, notice periods, and severance requirements. Higher values indicate greater labor market freedom.	459	54.807	13.642	20	92
Democracy Index	Mean of five V-Dem sub-indices (deliberative, egalitarian, electoral, liberal, and participatory democracy), ranging from 0 (absence of democracy) to 1 (high democracy).	460	0.313	0.16	0.047	0.662
Political Corruption	Composite index of corruption in executive, legislative, and judicial domains. Scores range from 0 (no corruption) to 1 (high corruption).	460	0.638	0.23	0.097	0.966

### Empirical modeling strategy

The baseline estimation relies on the Driscoll–Kraay standard errors, which are robust to serial correlation, heteroskedasticity, and cross-sectional dependence, issues commonly encountered in macro-panel data involving multiple countries over time. This correction enhances the reliability of statistical inference by accounting for spatial interconnectedness (such as regional spillovers) and temporal persistence (such as clustered shocks across years) that are typical features of African economies. To systematically evaluate the stability and robustness of the estimated coefficients, the model progressively adds control variables across specifications (2)–(7) in a stepwise sequence. The specification begins with the core variable of interest, climate finance, and then gradually introduces additional controls to identify how different sets of factors may influence the observed relationship.

Specifically, economic controls are introduced first, including economic development and population (log), to account for differences in macroeconomic capacity and scale effects across countries. Next, demographic variables, such as urbanization and the labor‑freedom index, are included to capture structural and labor‑market conditions that shape youth engagement. Finally, institutional and political factors, the democracy index, and political corruption are added in later models to reflect differences in governance quality and political environment. This gradual inclusion of controls ensures that the estimated association between climate finance and youth NEET outcomes is not confounded by underlying economic, demographic, or institutional differences and helps assess whether the main relationship remains robust under progressively richer model specifications.

1 $$\:{NEETS}_{it}={a}_{1}+{a}_{2}{CF}_{it}+{a}_{3}{X}_{it}+{v}_{i}+{{\upvarsigma\:}}_{t}+{{\upepsilon\:}}_{it\:}\:\:\:\:\:$$

Where:

- $\:{NEETS}_{it}$ denotes the Youth Employment Opportunity index for the country $\:i$ in year $\:t.$.
- $\:{CF}_{it}$ refers to total climate finance, respectively.
- $\:{X}_{it}$ represents the vector of control variables mentioned in Sect. 3.2.3.
- $\:{v}_{i}$ and $\:{{\upvarsigma\:}}_{t}$ are country and year fixed effects.
- $\:{{\upepsilon\:}}_{it\:}$ is the idiosyncratic error term.

Following the baseline estimations, an Instrumental Variables–Generalized Method of Moments (IV–GMM) estimation (Table 3) was implemented to address potential endogeneity in the relationship between climate finance and youth NEET rates. Endogeneity may arise from reverse causality, where countries with higher NEET levels attract more climate funding; omitted time‑varying factors such as institutional capacity or donor targeting criteria; and measurement errors in reported climate finance data. The IV–GMM specification mitigates these issues by using internal instruments derived from lagged regressors that exploit exogenous variation in climate finance while controlling for dynamic dependence within countries. Diagnostic tests support the validity and strength of the instruments: The Kleibergen–Paap and Cragg–Donald statistics exceed the Stock–Yogo critical values, indicating strong identification, and the Hansen J‑test confirms that the instruments are exogenous. Collectively, these results strengthen confidence that the estimated coefficients reflect a consistent and unbiased relationship between climate finance and youth NEET outcomes in the African sample.

Following the IV–GMM estimation, a comprehensive series of robustness checks was conducted to evaluate the stability, depth, and generalizability of the results. These tests explored whether the observed relationship between climate finance and youth NEET outcomes persisted under different model specifications, with expanded control sets, and across structurally diverse country groups.

## Results

### Baseline results

Table 2 presents baseline estimates examining the relationship between climate finance and youth NEET rates across 46 African countries from 2011 to 2021. The dependent variable, NEET, measures the percentage of individuals aged 15–24 years who are neither employed nor enrolled in education or training. The main explanatory variable, climate finance (log), is the natural logarithm of total climate‑related inflows, defined as the combined value of adaptation and mitigation finance (in thousands of USD) received annually by each country from public and non‑governmental sources. All models are estimated using Driscoll–Kraay standard errors, which correct for heteroskedasticity, serial correlation, and cross‑sectional dependence typical in macro‑panel data.

Across all seven model specifications, the coefficient on logged total climate finance is negative and statistically significant, indicating that higher levels of climate‑related funding are associated with lower youth NEET rates. In the fully specified model (Column 7), the coefficient of − 0.152, significant at the 10% level, implies that a 1% increase in total climate‑finance inflows is associated with an estimated 0.15% decrease in the share of young people not in education, employment, or training, holding other factors constant. Although the magnitude of the coefficient is moderate, it is economically meaningful given the scale of financing: for a country with a youth NEET rate of 30%, a 10% rise in climate‑finance inflows corresponds to an approximate 0.45‑percentage‑point reduction in NEETs. The consistency of the negative coefficient across all specifications suggests a stable relationship between climate‑related funding and youth‑employment outcomes. The control variables display the expected signs. Economic development and urbanization are negatively associated with NEET rates, whereas population growth is positively associated. Democracy is also negatively associated with NEET rates, while corruption is positively associated, consistent with findings that stronger institutions tend to coincide with better labor‑market outcomes. Overall, the baseline results indicate a statistically consistent negative association between climate finance and youth NEET rates across African countries, suggesting that expanded access to climate‑related financial resources is linked to improved youth employment and training participation over the study period.

**Table 2:** Baseline Result-Effect of CF on NEET -Driscoll & Kraay estimates.

	Dependent Variable: NEET						
	(1)	(2)	(3)	(4)	(5)	(6)	(7)
Climate finance (log)	−0.231***	−0.203*	−0.101***	−0.117**	−0.114**	−0.134**	−0.152*
	(0.0308)	(0.0983)	(0.0348)	(0.0564)	(0.0504)	(0.0628)	(0.0807)
Economic development		−0.990***	−0.0389	−0.848***	−0.666*	−0.802**	−1.094***
		(0.117)	(0.0950)	(0.200)	(0.312)	(0.300)	(0.320)
Population (log)			1.477***	1.338***	1.374***	1.239***	1.098***
			(0.0677)	(0.0590)	(0.0578)	(0.0814)	(0.0544)
Urbanization				−0.0857***	−0.0695***	−0.0588**	−0.0717***
				(0.0135)	(0.0165)	(0.0185)	(0.0205)
Labor Freedom Index					−0.0440**	−0.0570**	−0.0652**
					(0.0185)	(0.0199)	(0.0210)
Democracy Index						−9.229***	−5.072**
						(1.416)	(2.171)
Political Corruption							4.879***
							(0.956)
Constant	2.349***	1.622***	2.582***	2.817***	3.002***	2.910***	3.532***
	(0.856)	(1.201)	(0.785)	(0.836)	(0.835)	(0.833)	(1.898)
Observations	460	450	450	450	449	449	449
R-squared	0.222	0.236	0.296	0.315	0.419	0.444	0.453
Number of groups	46	46	46	46	46	46	46

Standard errors are in parentheses. *p* < 0.1 *, *p* < 0.05 **, *p* < 0.01 ***.

### IV-GMM estimates

To mitigate potential endogeneity, including reverse causality, omitted variable bias, and measurement error, the analysis uses an Instrumental Variables–Generalized Method of Moments (IV–GMM) estimation approach. Table 3 reports the results using lagged values of logged total climate finance as internal instruments to capture exogenous variation in climate‑related disbursements. The dependent variable is the NEET rate, measured as the percentage of individuals aged 15–24 years who are neither employed nor enrolled in education or training. The main explanatory variable, climate finance (log), represents the natural logarithm of total climate‑related inflows, defined as the combined value of adaptation and mitigation finance (in thousands of USD) received annually by each country from public and non‑governmental sources. Across all seven specifications (Columns 1–7), the coefficient on logged climate finance remains negative and statistically significant, consistent with the baseline results. In the fully specified model, the coefficient is − 0.231, significant at the 5% level, indicating that a 1% increase in total climate‑finance inflows corresponds to an estimated 0.23% point reduction in the youth NEET rate, holding other factors constant.

Diagnostic statistics support the adequacy of instruments. The Kleibergen–Paap rk Wald F‑statistics range from 13.15 to 25.56, exceeding the Stock–Yogo 10% critical value of 16.38 in most cases, indicating that the instruments are not weak. The Cragg–Donald F‑statistics also exceed conventional thresholds, confirming instrument relevance. The Hansen J‑test returns p‑values above conventional significance levels, indicating that the over‑identifying restrictions are satisfied and that the instruments are valid. Overall, the IV–GMM results provide consistent empirical evidence of a negative association between logged climate finance and youth NEET rates across African countries, even after controlling for potential endogeneity. These results are statistically robust, and the diagnostic tests indicate instrument strength and validity.

**Table 3:** IV-GMM estimates.

	Dependent Variable: NEET						
	(1)	(2)	(3)	(4)	(5)	(6)	(7)
Climate finance (log)	−0.352***	−0.707***	−0.531***	−0.678***	−0.484***	−0.915**	−0.231**
	(0.0523)	(0.0827)	(0.0378)	(0.0445)	(0.0379)	(0.556)	(0.0928)
Economic development		−1.373***	−1.344***	−1.435**	−1.303**	−1.012***	−1.867***
		(0.394)	(0.414)	(0.639)	(0.799)	(0.196)	(0.530)
Population (log)			0.333*	0.787***	0.263	0.275*	0.746***
			(0.183)	(0.266)	(0.215)	(0.112)	(0.231)
Urbanization				−0.0923**	−0.0884**	−0.0803	−0.106*
				(0.0386)	(0.0441)	(0.0501)	(0.0621)
Labor Freedom Index					−0.133***	−0.461***	−0.104**
					(0.0424)	(0.0582)	(0.0620)
Democracy Index						−1.353***	1.326*
						(0.346)	(0.891)
Political Corruption							2.039*
							(1.362)
Observations	414	405	405	405	404	404	404
Fisher (P-value)	0.000	0.000	0.000	0.000	0.000	0.000	0.000
Kleibergen-Paap rk Wald F	13.15	17.98	25.56	24.55	23.37	20.36	18.01
Cragg-Donald Wald F	21.89	10.75	39.58	38.50	37.31	32.46	28.53
Hansen J statistic	0	0	0	0	0	0	0
Stock-Yogo weak ID test critical values							
10% maximal IV size	16.38	16.38	16.38	16.38	16.38	16.38	16.38
15% maximal IV size	8.96	8.96	8.96	8.96	8.96	8.96	8.96
20% maximal IV size	6.66	6.66	6.66	6.66	6.66	6.66	6.66
25% maximal IV size	5.53	5.53	5.53	5.53	5.53	5.53	5.53

Standard errors are in parentheses. *p* < 0.1 *, *p* < 0.05 **, *p* < 0.01 ***.

### Additional covariates

Table 4 presents results from extended model specifications that incorporate additional socio‑structural covariates to assess the robustness of the relationship between climate finance and youth NEET rates. These models progressively introduce three variables, namely, the Education Index, the Discrimination Index, and Internet penetration, to account for broader human capital, social, and technological factors that may influence labor market outcomes. The dependent variable remains the NEET rate, defined as the percentage of individuals aged 15–24 who are neither employed nor enrolled in education or training. The main explanatory variable, climate finance (log), represents the natural logarithm of total adaptation and mitigation finance (in thousands of USD) received annually by each country from public and non‑governmental sources. Across all three specifications, the coefficient on logged climate finance remains negative and statistically significant, confirming the persistence of the baseline relationship under expanded model conditions. In Model (1), which includes the Education Index, the coefficient on climate finance is − 0.207 (significant at the 10% level), indicating that, even after controlling for differences in educational attainment across countries, higher climate‑finance inflows continue to correspond with lower youth NEET rates. In Model (2), after adding the Discrimination Index, which captures institutional and social constraints on labor-market participation, the coefficient remains negative (–0.131, significant at the 10% level), indicating that the association is not sensitive to measures of social inclusion or exclusion.

In Model (3), the addition of Internet penetration, a proxy for digital access and connectivity, strengthens the relationship further. The coefficient on logged climate finance increases in absolute value to − 0.486 and is significant at the 1% level. This indicates that the negative association between climate finance and youth NEET rates becomes more pronounced in economies with higher levels of technological access. The results in Table 4, therefore, show that the estimated effect of climate finance on youth NEET rates remains stable after controlling for educational quality, discrimination, and digital infrastructure. Overall, the evidence suggests that the observed relationship is robust to additional socio-economic controls and that higher levels of logged climate finance inflows consistently align with lower youth NEET rates across African countries.

**Table 4:** Additional Covariates.

	Dependent Variable: NEET		
	(1)	(2)	(3)
Climate finance (log)	−0.207*	−0.131*	−0.486***
	(0.111)	(0.0647)	(0.750)
Economic development	−1.093***	−0.622	−1.623***
	(0.286)	(0.532)	(0.431)
Population (log)	1.131***	1.075***	0.881***
	(0.0710)	(0.0834)	(0.0830)
Urbanization	−0.0561*	−0.0757**	−0.0980***
	(0.0250)	(0.0326)	(0.0235)
Labor Freedom Index	−0.0940***	−0.0779*	−0.00899
	(0.0247)	(0.0358)	(0.0208)
Democracy Index	−3.121	−1.022***	−2.809
	(2.352)	(0.329)	(2.063)
Political Corruption	4.175***	4.457***	3.719***
	(1.189)	(1.107)	(1.100)
Education Index	−0.0434***		
	(0.00626)		
Discrimination Index		0.521**	
		(0.189)	
Internet			−0.113***
			(0.00860)
Constant	3.683***	3.380***	3.823***
	(0.183)	(0.756)	(0.858)
Observations	409	439	449
R-squared	0.454	0.401	0.472
Number of groups	46	46	46

Standard errors are in parentheses. *p* < 0.1 *, *p* < 0.05 **, *p* < 0.01 ***.

### Disaggregation of climate finance variable

Table 5 presents results from models that disaggregate total climate finance into its two main components: adaptation finance and mitigation finance. This analysis tests whether the relationship between climate finance (log) and youth NEET rates is primarily associated with either form of climate-related investment. In Model (1), which includes logged adaptation finance, the coefficient is − 0.151 and statistically significant at the 1% level. Interpreted in log–linear terms, a 1% increase in adaptation finance corresponds to an estimated 0.15% decrease in the youth NEET rate, controlling for other variables.

In Model (2), where logged mitigation finance is included, the coefficient is − 0.198 and is significant at the 5% level, indicating that a 1% increase in mitigation finance is associated with an estimated 0.20% point reduction in the youth NEET rate. Both coefficients are negative and statistically significant, confirming that the inverse relationship between climate finance and youth inactivity holds when the overall measure is disaggregated into its constituent components. The magnitudes are similar, but the impact of mitigation finance appears slightly stronger. In general, the disaggregated estimates show that the negative link between logged climate finance inflows and youth NEET rates holds across all types of financing. These results indicate that both adaptation‑ and mitigation‑oriented climate investments are systematically linked to lower levels of youth inactivity across African countries, reinforcing the robustness of the overall relationship identified in earlier models.

**Table 5:** Disaggregation of Climate Finance.

	Dependent Variable: NEET	
	(1)	(2)
Adaptation finance (log)	−0.151***	
	(0.0448)	
Mitigation finance (log)		−0.198**
		(0.0830)
Economic development	−1.015**	−1.091**
	(0.319)	(0.338)
Population (log)	1.174***	0.991***
	(0.0535)	(0.0550)
Urbanization	−0.0696***	−0.0705***
	(0.0205)	(0.0206)
Labor Freedom Index	−0.0664***	−0.0642**
	(0.0199)	(0.0219)
Democracy Index	−4.964**	−5.417**
	(1.990)	(2.320)
Political Corruption	4.708***	4.975***
	(0.881)	(1.019)
Constant	3.446***	3.502***
	(0.771)	(0.770)
Observations	449	449
R-squared	0.447	0.460
Number of groups	46	46

Standard errors are in parentheses. *p* < 0.1 *, *p* < 0.05 **, *p* < 0.01 ***.

### Alternative dependent variables

To evaluate the robustness of the main findings, Table 6 reports estimations using two alternative dependent variables: vulnerable employment (% of total employment of youths and the Youth Employment Opportunity (YEO) index. The vulnerable employment measure follows the International Labor Organization’s definition, representing the share of own‑account and contributing family workers as a percentage of total youth employment, with higher values indicating greater job insecurity. The YEO index, derived from the Youth Development Index, ranges from 0 to 1, with higher scores indicating better youth employment opportunities. The main explanatory variable remains climate finance (log), defined as the natural logarithm of total adaptation‑ and mitigation‑related inflows (in thousands of USD) received annually by each country from both public and non‑governmental sources. In Model (1), the coefficient on logged climate finance is − 0.084 and statistically significant at the 1% level, indicating that a 1%-point increase in climate-finance inflows is associated with a 0.08%-point decline in vulnerable youth employment. In Model (2), the coefficient is 0.316 and significant at the 1% level, corresponding to an approximate 0.003‑point increase in the YEO index (0–1 scale). Across both models, the direction and significance of the coefficients remain consistent, confirming that higher logged climate‑finance inflows are associated with improved youth labor‑market outcomes.

**Table 6:** Alternative measures of NEET.

	Vulnerable Employment (% of total employment of Youths)	Youth Employment Opportunity
	(1)	(2)
Climate finance (log)	−0.0842***	0.316***
	(0.0186)	(0.0631)
Economic development	−0.478***	0.0179***
	(0.0257)	(0.00533)
Population (log)	0.0177**	−0.0204***
	(0.00663)	(0.00126)
Urbanization	−0.116*	0.104***
	(0.0565)	(0.00643)
Labor Freedom Index	−0.100***	0.177***
	(0.0164)	(0.0167)
Democracy Index	−0.285***	0.0452***
	(0.0474)	(0.00910)
Political Corruption	0.0169	−0.0360***
	(0.0373)	(0.00972)
Constant	3.392***	0.351***
	(0.162)	(0.0336)
Observations	449	451
R-squared	0.561	0.406
Number of groups	46	46

Standard errors are in parentheses. *p* < 0.1 *, *p* < 0.05 **, *p* < 0.01 ***.

### Heterogeneity analysis

Table 7 presents the heterogeneity analysis, which examines whether the relationship between climate finance and youth NEET rates differs by countries’ income levels. The sample is categorized into low-income and non-low-income African nations, in accordance with the World Bank’s (2025) income classification. In the low‑income group, the coefficient on logged climate finance is − 0.130 and statistically significant at the 5% level, indicating that a 1% point increase in climate‑finance inflows is associated with an estimated 0.13% point decrease in the NEET rate, holding other variables constant. In non‑low‑income countries, the coefficient is − 0.139 and significant at the 10% level, implying that a 1% increase in climate‑finance inflows corresponds to an approximate 0.14% reduction in NEETs.

Although the effect sizes are moderate, they are economically meaningful when scaled for realistic changes in financing levels. For example, a 10% rise in climate finance inflows, roughly US$40–60 million depending on country size, is associated with a 1.3–1.4% decline in youth NEET rates. The similarity in magnitude across income groups indicates that the observed relationship between climate finance and youth inactivity is broadly consistent regardless of economic classification. In both contexts, greater financial inflows coincide with lower shares of young people not engaged in education, employment, or training. These results reinforce the robustness of the baseline findings and suggest that the association persists across diverse structural conditions within African economies.

**Table 7:** Heterogeneity analysis.

	Low-Income African Countries	Non-Low-Income African Countries
	Dependent Variable: NEET	
	(1)	(2)
Climate finance (log)	−0.130**	−0.139*
	(0.0405)	(0.0814)
Economic development	−1.497***	−1.261*
	(0.291)	(0.908)
Population (log)	0.830***	0.738
	(0.102)	(1.368)
Urbanization	−0.0309	−0.423***
	(0.0295)	(0.0294)
Labor Freedom Index	−0.147***	−0.270***
	(0.0256)	(0.0734)
Democracy Index	−3.581*	0.101
	(1.609)	(0.288)
Political Corruption	1.377***	1.444***
	(0.202)	(0.124)
Constant	3.092***	−2.231***
	(0.866)	(0.794)
Observations	373	76
R-squared	0.460	0.527
Number of groups	38	8

Standard errors are in parentheses. *p* < 0.1 *, *p* < 0.05 **, *p* < 0.01 ***.

### Overall findings

Across all model specifications, climate finance consistently exhibits a negative and statistically significant relationship with youth NEET rates in African countries, indicating that higher inflows of climate‑related funding are associated with lower levels of youth disengagement from education, employment, or training. The results remain robust when addressing endogeneity through IV–GMM estimation, incorporating additional social and technological covariates, and testing alternative measures of youth employment outcomes. Moreover, disaggregated and heterogeneity analyses confirm that this relationship holds across different forms of climate finance and country income groups, underscoring the stability, consistency, and strength of the link between climate‑finance inflows and reduced youth labor‑market vulnerability across the region.

## Discussion and conclusion

### Reasons for the results

This study examined whether climate‑finance inflows are associated with lower youth NEET rates across African countries, assessing the extent to which external environmental funding supports human‑capital development and labor‑market inclusion. By noting climate finance as a developmental rather than purely ecological instrument, the paper provides cross-national evidence on how global climate investments intersect with youth labor systems. Drawing on panel data for 46 countries from 2011 to 2021 and rigorous empirical methods, the analysis finds a consistent negative association between climate‑finance inflows and youth NEET rates, consistent with H₁, thereby linking climate investment to broader processes of human‑capital enhancement and inclusive economic participation.

The findings align with the central proposition of Human Capital Theory, which posits that education, skills, and knowledge are productive assets that expand both individual and societal capacity for adaptation and growth^13,14^. From this perspective, climate finance functions as an external source of investment that augments a country’s stock of human capital. As climate‑related resources flow into skill‑intensive sectors, they indirectly finance learning, technological mastery, and institutional capability. These processes improve the efficiency and quality of human capital within recipient economies, raising labor productivity and enabling structural transformation^34,54^. In line with HCT, economies that increase such investments can expect higher returns in the form of broader labor absorption and lower inactivity, as the upgraded skill base reduces structural bottlenecks that often limit employment creation for new entrants. Comparable evidence from other regions supports this pattern: in China, for instance, green‑finance frameworks have been found to foster industrial upgrading and generate longer‑term employment gains^33,34^. These parallels suggest that the developmental effects of climate finance extend beyond environmental outcomes to tangible gains in employment and capability formation when institutional systems effectively absorb external resources. However, it stands in contrast to findings by Zhao, Li, and Hou^36^ who report that mitigation‑focused projects can exacerbate gender disparities in developing economies, particularly in Africa, where capital‑intensive green sectors remain male‑dominated and social norms restrict women’s participation in climate‑related employment.

Among African youth, the effects of enhanced human capital accumulation are particularly salient. Young Africans often face entry barriers rooted in weak vocational systems, limited practical experience, and the scarcity of skill‑relevant opportunities^41^. When climate‑finance inflows strengthen the systems that generate and utilize skills, they improve both the supply-side capabilities and demand‑side readiness necessary for youth participation. Through this investment logic, climate finance reinforces the HCT premise that productivity gains from education and training translate into higher returns to employability and participation^39^. A more skilled and adaptable youth cohort is therefore better positioned to transition from education or inactivity into gainful employment, leading to a measurable decline in NEET rates. Although this study examines associations rather than causal mechanisms, the results consistently support the theoretical expectation that externally financed investments in human capital contribute to greater youth inclusion in education, training, and productive work across African economies.

Beyond potential climate‑finance influence, the results also point to a broader set of economic, demographic, and institutional conditions that shape NEET rates and determine how effectively such external investments translate into opportunities for youth. The associations observed for the control variables are consistent with these broader forces. The negative link between economic development and NEET rates suggests that as economies grow and diversify, they generate more productive forms of employment and strengthen the systems that connect education to work. When overall income levels rise, governments and firms alike have greater capacity to invest in training, vocational education, and industrial expansion, helping young people transition into the labor market^47^. By contrast, limited development and heavy dependence on informal or subsistence activities constrain job creation and reduce access to skill‑building opportunities, leaving many young people without viable employment pathways. The positive association with population growth reflects the demographic pressure created by Africa’s expanding youth cohort: when labor-market absorption or training systems fail to keep pace with population growth, competition for jobs intensifies, and a larger share of young people remain disengaged^48^. Meanwhile, the negative effects of urbanization point to the benefits of spatial concentration. Urban areas typically provide greater access to schools and technical colleges, as well as a more diverse range of occupations, all of which enhance labor‑market connections and lower NEET rates^49,50^.

Institutional quality further shapes the effectiveness with which young people are integrated into the labor market. The negative coefficient for the Labor Freedom Index indicates that flexible regulations and a supportive business environment make it easier for firms to hire and retain workers, opening entry points for youth employment^51^. The similarly negative relationship for the Democracy Index suggests that inclusive and accountable governance helps sustain education and training initiatives that prepare youth for available opportunities^52^. In contrast, the positive link between political corruption and NEET rates reveals how weak institutions and rent-seeking practices undermine these processes by eroding trust, distorting investment priorities, and diverting funding away from employment-oriented programs^53^. Altogether, these findings show that improving youth economic participation requires not only climate-related financial inflows but also a conducive domestic environment in which economic structure, demographic management, and institutional integrity enable such resources to become genuine opportunities for education, training, and work.

### Policy implications

The results point to meaningful opportunities for African governments, regional institutions, and development partners to align climate finance more closely with the continent’s employment and human‑capital priorities. The consistent negative relationship between higher climate‑finance inflows and youth NEET rates indicates that when effectively absorbed, these funds can do more than finance environmental resilience. They can stimulate local skill development, improve access to training, and expand pathways into the workforce for Africa’s rapidly growing youth population. In a region where formal employment remains scarce and informality dominates, the developmental value of climate finance lies in its potential to strengthen the systems that connect young people to productive and adaptive livelihoods.

At the national level, the findings underscore the need to view climate finance not as a separate environmental instrument but as part of Africa’s broader development architecture. Collaboration among ministries of finance, labor, education, and environment is essential to explicitly design climate-related projects that generate youth-inclusive benefits. Linking renewable‑energy programs with technical training in solar installation, embedding skills‑transfer components within sustainable agriculture projects, or coupling watershed and infrastructure investments with community‑based apprenticeships are practical ways to maximize employment gains. In many African countries, bureaucratic inefficiencies and limited absorptive capacity remain major barriers; streamlining procedures, enhancing accountability, and improving coordination across ministries would increase the likelihood that external inflows translate into real jobs and training opportunities. The evidence that even modest inflows correspond to lower NEET rates suggests that impact depends less on volume and more on institutional readiness, a crucial insight for low‑income African economies, where capacity constraints are most acute.

For regional organizations and international partners, the challenge is to strengthen the enabling environment for climate‑finance absorption while tailoring strategies to Africa’s structural context. Expanding financial commitments without building administrative or technical capacity risks perpetuating dependence rather than deepening resilience. Climate‑finance frameworks that incorporate technical assistance, governance support, or results‑based incentives could help ensure that funds achieve both environmental and social outcomes. Regional knowledge‑exchange platforms, facilitated by bodies such as the African Development Bank or the African Union, could document cases where climate‑related programs have successfully expanded employment, from Rwanda’s green‑jobs initiatives to Kenya’s off‑grid renewable‑energy enterprises. Such initiatives would enable peer learning and replication to be adapted to differing institutional capacities and labor-market conditions across the continent.

Civil‑society organizations and the private sector also have essential roles in localizing and sustaining these outcomes. NGOs can identify community-specific barriers to youth participation, design inclusive training programs, and monitor whether climate investments benefit vulnerable populations rather than reinforcing existing inequalities. For private investors, particularly in sectors such as agribusiness, renewable energy, and infrastructure, integrating training clauses, youth employment targets, and gender-responsive hiring practices into project frameworks can extend the reach of climate-finance benefits. In contexts where informality prevails and labor protections are limited, public‑private partnerships that create structured pathways into green employment are especially valuable. Taken together, the study’s results suggest that reducing youth NEET rates through climate finance depends not only on financial inflows but also on the institutional and policy environment in which those funds operate. Aligning external resources with Africa’s national education, training, and employment systems can transform climate finance from a source of project funding into a driver of inclusive growth. By reinforcing vocational capacity, improving governance, and nurturing labor‑absorptive economic sectors, African countries can harness climate finance as both a climate‑adaptation tool and a catalyst for a more resilient and youth‑inclusive development trajectory.

### Limitations and future research direction

This study contributes new cross-national evidence on the relationship between climate finance and youth NEET rates in Africa, but it also faces several limitations that suggest directions for future research. First, the temporal scope is confined to 2011–2021, a period that captures the maturation of climate‑finance mechanisms but not the structural shifts of the post‑COVID‑19 era. The economic dislocations of the pandemic and the geopolitical tensions of the early 2020 s have altered donor priorities, trade patterns, and fiscal space across African economies, potentially reshaping both the scale of climate‑finance inflows and their developmental impacts. Future studies should therefore extend the temporal frame to incorporate the post‑2021 context, examining how new frameworks, such as just‑transition financing, carbon‑market initiatives, and green‑industrial strategies, affect youth employment and human‑capital resilience in a more uncertain global economy.

Second, the geographic focus on Africa enhances theoretical coherence but limits external comparability. Africa’s rapid population growth, high levels of informality, and limited institutional capacity make it a unique empirical setting. Nevertheless, similar challenges, such as youth underemployment, governance fragility, and vulnerability to climate shocks, are emerging in other developing regions. Comparative analyses across Latin America, South Asia, and Small Island Developing States could test whether the employment effects observed here reflect a continental pattern or are part of a broader structural relationship between climate finance and inclusive growth. Such cross‑regional work could also potentially identify region‑specific institutional models that explain variation in effectiveness.

Third, despite the use of robust estimation techniques, including IV-GMM, to address potential endogeneity, the analysis remains correlational rather than causal. Establishing a clear causal link between climate finance inflows and youth labor outcomes is challenging because funding allocations may themselves respond to economic or social conditions and because the channels of influence are indirect and multifaceted. Future research could move beyond aggregate relationships by exploiting quasi‑experimental or natural‑experiment conditions, such as donor‑allocation thresholds, program eligibility rules, or staggered rollouts of large climate initiatives, to identify exogenous variation in financial inflows. More refined causal approaches could also examine how different types of investment, such as renewable‑energy expansion, sustainable‑agriculture programs, or climate‑resilient infrastructure, affect employment across economic sectors with varying labor intensities. For example, investing in agriculture or restoring ecosystems may create jobs right away for young people in rural areas, while low-carbon industrial or energy projects may take longer to create jobs but offer more technical, skill-intensive work.

Fourth, while the analysis focuses on youth as a single demographic group, this approach overlooks critical heterogeneity within the population. Differences by gender, socio‑economic status, education level, and geographic location, particularly between urban and rural contexts, may shape both access to climate‑finance‑related opportunities and the capacity to benefit from them. Treating youth as a homogeneous category masks these disparities and may obscure how climate‑finance inflows translate into employment gains or exclusions for specific subgroups. The impacts of climate finance may also vary across younger and older youth cohorts, reflecting differences in educational attainment, work experience, and exposure to training programs. Future research should therefore adopt a more disaggregated approach. Subgroup analyses that examine intersections of gender, income, age, and spatial context could reveal how climate-related investments interact with pre-existing inequalities in education, labor markets, and social capital. For example, women and young people living in rural areas may have a harder time getting into green economy training programs or formal job networks. This could mean that even when the overall NEET rate goes down, the benefits are not evenly distributed. Such differentiation would improve the precision of causal inference and yield policy insights into the kinds of climate‑finance interventions, whether in renewable energy, agriculture, or infrastructure, that most effectively enhance inclusion and equity among Africa’s diverse youth populations.

Fifth, the study abstracts from potential negative externalities and moderate dynamics in the nexus between climate‑finance inflows and NEET rates. The analysis adopts a linear regression framework that captures statistical associations but does not trace the pathways through which climate finance influences youth inclusion, nor identify the contextual conditions under which these effects occur. Institutional quality, both political and economic, may critically mediate whether financial inflows translate into meaningful opportunities or instead reinforce existing inequalities. While the results suggest an overall positive association with youth participation, poorly designed or weakly monitored projects could also generate adjustment costs, including labor displacement, elite capture, or environmental degradation. Future work should therefore move beyond estimating average associations by employing non-linear, interaction, or mediation models that explicitly incorporate moderating/mediating variables such as political governance, institutional performance, inequality, and sectoral dependence. These approaches could help uncover the mechanisms and contingencies through which climate finance fosters inclusive growth in some settings but fails or backfires in others. In other words, while this study provides macro‑level evidence of association, it leaves the underlying causal architecture—the “black box” connecting financial inflows, institutional behavior, and employment outcomes—largely unexplored.

Sixth, although the NEET indicator offers a standardized, cross‑national measure of youth inactivity, it inevitably conceals substantial variation in the quality, stability, and nature of employment among Africa’s youth. Individuals classified as “not in employment, education, or training” represent diverse groups, from discouraged job seekers and recent school leavers to unpaid family workers, each experiencing distinct labor-market constraints and livelihood strategies. Moreover, many African youths work in informal or subsistence economies that contribute to their livelihoods, but conventional labor statistics do not capture this work. Consequently, the binary NEET measure effectively identifies exclusion from formal education or employment systems but not the broader continuum of informal, low‑quality, or precarious work that typifies much of Africa’s labor market. Future research could address these limitations by complementing cross-national NEET data with multidimensional indices of youth employment that capture job quality, income security, skill utilization, and working conditions once such data become available. Integrating these indicators with micro-level labor-force or household survey datasets would facilitate disaggregation by gender, income, and geography, thereby aiding in the differentiation between inactivity, underemployment, and informal work. Linking these micro‑level datasets with project‑level or sector‑specific climate‑finance information would further illuminate the causal transmission channels through which climate investment affects occupational mobility, skills development, and labor‑market transitions. Taken together, these extensions would close part of the current “black box” between financial inflows and social outcomes, offering richer insights into how climate finance translates into meaningful and sustainable youth employment across diverse African contexts.

## Data Availability

The raw data supporting the findings of this study are available from the corresponding author upon reasonable request.

## Appendix

**Table:** List of African Countries Included in This Study

Angola	Egypt	Libya	Senegal
Benin	Equatorial Guinea	Madagascar	Seychelles
Botswana	Eritrea	Mali	Sierra Leone
Burkina Faso	Ethiopia	Mauritania	Swaziland
Burundi	Gabon	Mauritius	Tanzania
Cameroon	Gambia	Morocco	Togo
Cape Verde	Ghana	Mozambique	Tunisia
Central African Republic	Guinea	Namibia	Uganda
Chad	Guinea-Bissau	Nigeria	Zambia
Comoros	Ivory Coast	Republic of the Congo	Zimbabwe
Democratic Republic of the Congo	Kenya	Rwanda	
Djibouti	Liberia	São Tomé and Principe	

## References

[1] Kelly, A. M. & Wassou, N. L. Climate change and its effect on health outcomes: An empirical investigation of Sub-Saharan Africa countries. *SN Soc. Sci.* **5**(4), 1–19. 10.1007/s43545-025-01081-1 (2025).

[2] Kelly, A. M. Does climate finance foster happiness in African economies? Assessing the direct and indirect pathways. *Glob. Transit.* **7**, 310–322. 10.1016/j.glt.2025.05.003 (2025).

[3] Ko, J., Leung, C. K., Dodoo, A. N. & Agbajor, F. D. Regional disparities and trends in national ESG performance across Africa: A comparative analysis (1990–2020). *Innov. Green Dev.* **4**(3), 100231. 10.1016/j.igd.2025.100231 (2025).

[4] Gemayel, E. R., Rosa, S., Maheshwari, V., Ungerer, C. & Lindner, P. Laying the Ground for Scaling up Climate Finance in Sub-Saharan Africa. *IMF Working Papers*. **2025(099)**10.5089/9798229004091.001.A001 (2025).

[5] African Development Bank. *Action plan on climate change*. African Development Bank Group. (2022). https://www.afdb.org/en/topics-and-sectors/sectors/climate-change/action-plan-climate-change

[6] *Investing in Youth, Transforming Africa*. World World Bank & Bank (2023)., June 27 https://www.worldbank.org/en/news/feature/2023/06/27/investing-in-youth-transforming-afe-africa

[7] International Labour Organization. *Employment trends for youth in Sub-Saharan Africa*. International Labour Organization. (2024)., August 9 https://www.ilo.org/publications/employment-trends-youth-sub-saharan-africa

[8] Ibourk, A. & Elouaourti, Z. Youth ‘not in education, employment, or training’ (NEET) in North Africa: Do household structure, subjective factors, and political dimensions affect NEET outcomes differently by gender?. *J. Educ. Work.* **37**(7–8), 613–633. 10.1080/13639080.2025.2534786 (2024).

[9] Doku, I., Ncwadi, R. & Phiri, A. Determinants of climate finance: Analysis of recipient characteristics in Sub-Saharan Africa. *Cogent Econ. Financ.* **9**(1), 1964212. 10.1080/23322039.2021a.1964212 (2021aa).

[10] Aquilas, N. A. & Atemnkeng, J. T. Climate-related development finance and renewable energy consumption in greenhouse gas emissions reduction in the Congo Basin. *Energy Strategy Rev.* **44**, 100971 (2022).

[11] Buchner, B. et al. Global landscape of climate finance 2019. *Climate Policy Initiative* **32**(1), 1–38 (2019).

[12] Doku, I. Are developing countries using climate funds for poverty alleviation? Evidence from Sub-Saharan Africa. *Eur. J. Dev. Res.* **34**(6), 3026–3049. 10.1057/s41287-022-00509-1 (2022).

[13] Schultz, T. W. Investment in Human Capital. *Am. Econ. Rev.* **51** (1), 1–17 (1961).

[14] Becker, G. S. *Human Capital: A Theoretical and Empirical Analysis, with Special Reference to Education.* Second edition, University of Chicago Press. (1964).

[15] Lucas, R. E. On the mechanics of economic development. *J. Monet. Econ.* **22**(1), 3–42. 10.1016/0304-3932(88)90168-7 (1988).

[16] Romer, P. M. Endogenous technological change. *J. Polit. Econ.* **98**(5, Part 2), S71–S102. 10.1086/261725 (1990).

[17] Mincer, J. The production of human capital and the life cycle of earnings: Variations on a theme. *J. Labor Econ.* **15**(1, Part 2), S26–S47. 10.1086/209855 (1997).

[18] Psacharopoulos, G. Returns to investment in education: A global update. *World Dev.* **22**(9), 1325–1343. 10.1016/0305-750X(94)90007-8 (1994).

[19] Joshi, A. & Pandey, A. Advancing Climate Finance for Sustainable Future: Integrating Human Capital, Climate Neutrality, and Emerging Technologies. In R. Singh & D. Crowther (Eds.), *Climate Neutrality and Sustainable Ecosystems: Practical Approaches to Addressing Global Warming* (pp. 231–246). Springer Nature. (2025). 10.1007/978-981-96-7181-6_13

[20] Tsomb, E. I. B. T. When Does Climate Finance Foster Green Innovation? The Role of Human Capital Thresholds in Developing Countries. *Sustain. Dev.* **n/a** (n/a). 10.1002/sd.70962 (n.d.).

[21] Phiri, A. & Doku, I. Is climate finance aiding food security in developing countries? A focus on Sub-Sahara Africa. *Cogent Econ. Financ.* **12**(1), 2312777. 10.1080/23322039.2024.2312777 (2024).

[22] Doku, I., Richardson, T. E. & Essah, N. K. Bilateral climate finance and food security in developing countries: A look at German donations to Sub-Saharan Africa. *Food Energy Secur.* **11**(3), e412. 10.1002/fes3.412 (2022).

[23] Doku, I., Ncwadi, R. & Phiri, A. Examining the role of climate finance in the environmental kuznets curve for Sub-Saharan African countries. *Cogent Econ. Financ.* **9**(1), 1965357. 10.1080/23322039.2021b.1965357 (2021bb).

[24] Lee, C.-C., Li, X., Yu, C.-H. & Zhao, J. The contribution of climate finance toward environmental sustainability: New global evidence. *Energy Econ.* **111**, 106072. 10.1016/j.eneco.2022.106072 (2022).

[25] Gong, X., Borojo, D. G. & Yushi, J. The heterogeneous impacts of climate finance on environmental sustainability and social welfare in developing countries. *Kybernetes* **54**(3), 1904–1937. 10.1108/K-05-2023-0839 (2023).

[26] Kelly, A. M. Battling for food security in Africa: Is climate finance the missing bullet?. *World Food Policy* **10**(2), 227–253. 10.1002/wfp2.12078 (2024).

[27] Borojo, D. G., Yushi, J., Gong, X., Zhang, H. & Miao, M. The heterogeneous impacts of climate finance on energy efficiency and renewable energy production in developing countries. *Renew. Energy* **236**, 121427. 10.1016/j.renene.2024.121427 (2024).

[28] Njangang, H., Padhan, H. & Tiwari, A. K. From aid to resilience: Assessing the impact of climate finance on energy vulnerability in developing countries. *Energy Econ.* **134**, 107595. 10.1016/j.eneco.2024.107595 (2024).

[29] Wang, M. & Njangang, H. The unexpected outcomes: How does climate finance affect corruption in developing countries?. *J. Environ. Manage.* **389**, 126065. 10.1016/j.jenvman.2025.126065 (2025). 40480109 10.1016/j.jenvman.2025.126065

[30] Wong, S. Can climate finance contribute to gender equity in developing countries?. *J. Int. Dev.* **28**(3), 428–444. 10.1002/jid.3212 (2016).

[31] Zhao, J., Li, X. & Hou, C. Can climate finance bridge the gender gap in labor markets? Evidence from developing countries. *Clim. Change*. **178** (4), 80. 10.1007/s10584-025-03921-8 (2025).

[32] Mohan, P. & Morris, D. Climate finance spillovers and entrepreneurship in developing countries. *Strateg. Entrepreneurship J.* **18** (3), 475–501. 10.1002/sej.1517 (2024).

[33] Xin, B., Zhou, L. & Santibanez Gonzalez, E. D. R. Does green finance reform hit urban employment? — Evidence from China’s green finance pilot policy. *Cities* **152**, 105239. 10.1016/j.cities.2024.105239 (2024).

[34] Fu, B., Zhang, Y., Maani, S. & Wen, L. Green finance and job creation: Analyzing employment effects in China’s manufacturing industry within green finance innovation and reform pilot zones. *Energy Econ.* **141**, 108090. 10.1016/j.eneco.2024.108090 (2025).

[35] Rutazihana, P. N. & Kelly, A. M. Climate finance and natural resource depletion in Africa: Does institutional quality matter?. *Soc. Nat. Resour.* **0**(0), 1–25. 10.1080/08941920.2025.2553354 (2025).

[36] Zhao, J., Wang, J. & Dong, K. The role of green finance in eradicating energy poverty: Ways to realize green economic recovery in the post-COVID-19 era. *Econ. Change Restruct.* **56**(6), 3757–3785. 10.1007/s10644-022-09411-6 (2023).

[37] Li, X., Yu, C. H., Zhao, J., Lee, C. C. & Li, X. Achieving climate justice: Climate finance and income inequality in developing countries. *Clim. Dev.* **0**(0), 1–17. 10.1080/17565529.2026.2633559 (2026).

[38] Xu, W., Wang, X., Zhang, Y. & Wang, F. Impact of green finance on poverty reduction: Evidence from China. *Environ. Res. Commun.* **7**(1), 015015. 10.1088/2515-7620/ada734 (2025).

[39] Aleksandrova, M., Kuhl, L. & Malerba, D. Unlocking climate finance for social protection: An analysis of the Green Climate Fund. *Clim. Policy*. **24** (7), 878–893. 10.1080/14693062.2024.2338817 (2024).

[40] Nor, M. I. Exploring the nexus between climate finance, rural–urban disparities, and rural brain drain in Somalia: The mediating role of climate resilience. *Front. Sociol.*10.3389/fsoc.2025.1702333 (2025). 41268194 10.3389/fsoc.2025.1702333 PMC12626978

[41] Baah-Boateng, W. The youth unemployment challenge in Africa: What are the drivers?. *Econ. Labour Relat. Rev.* **27**(4), 413–431. 10.1177/1035304616645030 (2016).

[42] Olubusoye, O. E., Salisu, A. A. & Olofin, S. O. Youth unemployment in Nigeria: Nature, causes and solutions.. *Qual. Quant.* **57**(2), 1125–1157. 10.1007/s11135-022-01388-8 (2023).

[43] Dodoo, A. N., Ko, J., Ma, Y. & Guo, C. Revisiting sustainable development in Africa through a national economic environmental social and governance perspective.. *Discov. Sustain.* **6**(1), 1426. 10.1007/s43621-025-02079-8 (2025).

[44] Momo, M. S. M., Cabus, S. J., De Witte, K. & Groot, W. A systematic review of the literature on the causes of early school leaving in Africa and Asia.. *Rev. Educ.* **7**, 496–522. 10.1002/rev3.3134 (2019).

[45] Nilsson, B. The school-to-work transition in developing countries.. *J. Dev. Stud.* **55**(5), 745–764. 10.1080/00220388.2018.1475649 (2019).

[46] Hong, C., Liu, N. & Kelly, A. M. Can Climate Finance Improve Agricultural Productivity? Evidence From African Countries. *Sustain. Dev.*10.1002/sd.70793 (2026).

[47] Roemer, J. E. Economic development as opportunity equalization.. *World Bank Econ. Rev.* **28**(2), 189–209. 10.1093/wber/lht023 (2014).

[48] Weber, H. Age structure and political violence: A re-assessment of the youth bulge hypothesis. *Int. Interact.* **45** (1), 80–112. 10.1080/03050629.2019.1522310 (2019).

[49] Song, J., Du, H. & Li, S. Mobility and life chances in urbanization and migration in China: Introduction. *China Review* **18**(1), 1–10 (2018).

[50] Chen, M., Huang, X., Cheng, J., Tang, Z. & Huang, G. Urbanization and vulnerable employment: Empirical evidence from 163 countries in 1991–2019. *Cities* **135**, 104208. 10.1016/j.cities.2023.104208 (2023). 36777788 10.1016/j.cities.2023.104208 PMC9897722

[51] Das, N. & Kapoor, R. Assessing labour freedom in agriculture: Developing world perspective focusing on India. *J. Public Aff.* **24**(2), e2914. 10.1002/pa.2914 (2024).

[52] Deacon, R. T. Public good provision under dictatorship and democracy. *Public Choice* **139**(1), 241–262. 10.1007/s11127-008-9391-x (2009).

[53] Oueghlissi, R. & Derbali, A. Democracy, corruption and unemployment: Empirical evidence from developing countries. *J. Knowl. Econ.* **15**(2), 7475–7496. 10.1007/s13132-023-01204-0 (2024).

[54] Mustapha, A. & Palladan, A. A. Integrating green skills capacity building for a climate-responsive workforce: A comprehensive review of development finance institutions’ support towards achieving SDGs. *Sustain. Dev.* **34**(S2), 966–978. 10.1002/sd.70380 (2026).

